# Relationship of early pregnancy maternal plasma progesterone concentrations on piglet birth weight, within-litter birth weight variation, and piglet mortality from crossbred Landrace or Yorkshire dams bred with purebred Landrace or Yorkshire sires

**DOI:** 10.1093/jas/skaf415

**Published:** 2025-12-03

**Authors:** Jeremy R Miles, Clay A Lents, Robert A Cushman, Gary A Rohrer, Lea A Rempel

**Affiliations:** U.S. Department of Agriculture (USDA), U.S. Meat Animal Research Center, Clay Center, NE 68933; U.S. Department of Agriculture (USDA), U.S. Meat Animal Research Center, Clay Center, NE 68933; U.S. Department of Agriculture (USDA), U.S. Meat Animal Research Center, Clay Center, NE 68933; U.S. Department of Agriculture (USDA), U.S. Meat Animal Research Center, Clay Center, NE 68933; U.S. Department of Agriculture (USDA), U.S. Meat Animal Research Center, Clay Center, NE 68933

**Keywords:** birth weight, farrowing traits, pigs, preweaning mortality, progesterone, within-litter variation

## Abstract

As litter size in commercial U.S. swine herds has increased over recent decades, preweaning mortality (PWM) of piglets has also risen, driven in part by reduced piglet birth weight (BW) and greater within-litter BW variation. This study evaluated whether natural variation in maternal concentrations of plasma progesterone (P4) during early gestation influences key litter traits including piglet BW, within-litter BW variation, and PWM in crossbred Landrace- or Yorkshire-sired dams bred to purebred sires of the same breeds. A total of 1,535 pregnancies were analyzed across 4 parities, with plasma P4 collected at day 7 of gestation, quantified via radioimmunoassay, and evaluated using Pearson correlation analysis with key farrowing and weaning outcomes. Evaluation of the influence of dam and sire genetics as well as parity on these key litter traits were further analyzed using a MIXED model. Overall, early gestational P4 showed a weak (*r* = ±0.05) but significant (*P *< 0.05) positive relationship for litter size and a negative relationship for piglet BW, particularly in Yorkshire-sired dams. However, no direct associations were found between P4 and within-litter BW variation or PWM. Interestingly, Landrace-sired litters had greater P4 concentrations, greater BW, and lower PWM compared to Yorkshire-sired litters (*P *< 0.05), irrespective of dam genetics. This suggests a paternal influence on uterine environment that provides an indirect positive relationship between early gestational P4 and PWM. More consistent and biologically relevant relationships (ranging between *r* > −0.29 and <0.88) were observed between PWM and key farrowing traits. Significant (*P *< 0.01) parity effects were observed in which third and fourth parity dams had larger litters with reduced BW, increased within-litter variation, and greater PWM, while second parity dams had the largest piglet BW and lowest PWM. Furthermore, reciprocal crossbred matings yielded significantly (*P* < .01) lower BW variation and PWM compared to intra-line matings, indicating heterosis benefits, although these data are likely confounded by parity. These findings suggest that, rather than focusing on P4 variation alone, future management and genetic selection strategies should prioritize enhancing fetal growth and uniformity by optimizing parity structure and maximizing the benefits of strategic crossbreeding to sustain improvements in piglet quality and survival.

## Teaser

Larger litters in U.S. swine herds have increased piglet mortality before weaning, primarily due to smaller and less uniform piglet birth weights. This study found that sire genetics, sow parity, and crossbreeding have a much greater impact on piglet size and survival than early gestation progesterone levels alone, pointing to parity management and strategic crossbreeding as key strategies for improved piglet quality and survival.

## Graphical abstract

**Graphical Abstract:**
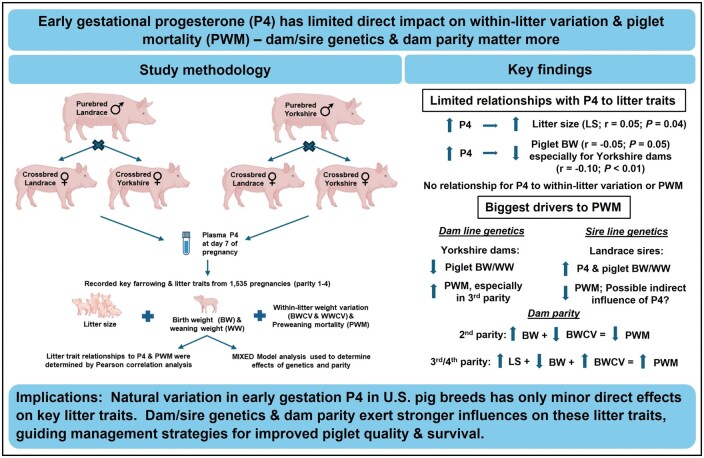

## Introduction

As the swine industry has selected for increased litter size over the past 2 decades, there has been a consequential increase in preweaning piglet mortality rates. During this time, average litter size in commercial U.S. herds have increased from 10.3 to 13.5 piglets born alive (PBA); however, during this same period preweaning mortality (PWM) rates have increased from 12.5% to 15.4% (Miles et al., 2023). Given that litter size significantly influences prenatal development and has profound effects on birth weight (BW) (Foxcroft et al., 2009), increasing litter size has a significant negative impact on BW, particularly within-litter birth weight variation and the production of smaller piglets, which plays a key role in subsequent PWM (van der Lende et al., 2001; Damgaard et al., 2003; Kemp et al., 2018). Therefore, increasing the comprehensive understanding of mechanisms that influence litter size, BW, within-litter BW variation, and PWM have significant potential to develop strategies to improve piglet quality and weaning survivability of all littermates.

Litter size is influenced by several factors including ovulation rate (OR), fertilization rate, embryonic mortality (both early embryonic and fetal), and uterine capacity (Vallet, 2000). As the swine industry has selected for PBA, there has been an indirect selection for OR, given that OR is a key component of litter size and both have increased during selection for PBA (Da Silva et al., 2018; Kemp et al., 2018). During the estrous cycle and early pregnancy, ovulated follicles develop into individual corpora lutea (CL) (Hafez, 1993). The CL produces and secretes progesterone (P4), which is critical for priming the uterine environment for the establishment and maintenance of pregnancy (Hafez, 1993). As OR increases in pigs, there is an increase in circulating P4 as demonstrated by Knox and colleagues when evaluating gilts with high OR and elevated P4 compared with low OR and reduced P4 (Knox et al., 2003). This difference in P4 was apparent by day 3 of the estrous cycle and maintained throughout the estrous cycle (Knox et al., 2003).

Although P4 is necessary for priming the uterine environment during early pregnancy (Geisert and Schmitt, 2002), evidence using an asynchronous embryo transfer model in cattle has demonstrated that high P4 concentrations during early pregnancy influenced embryonic development of conceptus length and increased variation in conceptus length when utilizing multiple embryos transferred into dams (Lonergan et al., 2016; Randi et al., 2016). Similarly in the pig, gilts treated with exogenous P4 during early pregnancy (day 3) had increased conceptus diameter at day 11 of pregnancy (Vallet and Christenson, 2004) and increased uterine protein secretion, retinol binding protein and uteroferrin between day 10 and 15 of pregnancy during critical conceptus elongation and subsequent superficial implantation (Vallet et al., 1998). These findings suggest that elevated P4 during early pregnancy may advance the uterine environment such that slight variation between embryos in cell cycle progression potentially result in asynchronous initiation of elongation and provide a possible mechanism for greater within-litter BW variation as litter size has increased.

Currently, it is not known whether natural variation in the levels of P4 during early gestation influences subsequent farrowing and weaning success in commercial maternal line dams in the U.S. Therefore, the objective of this study was to determine if plasma P4 levels during early pregnancy influences BW, within-litter BW variation, piglet mortality, and lactational growth. Furthermore, this study evaluated whether any influences of P4 and corresponding farrowing and weaning success are affected by sow parity as well as dam and sire genetic background between commercial Landrace and Yorkshire pigs. We hypothesize that increased P4 during early pregnancy will have a negative impact on BW, within-litter BW variation, and piglet weaning survival.

## Materials and Methods

### Animal populations, breeding, and gestation

All animal protocols were approved by the U.S. Meat Animal Research Center (USMARC) Animal Care and Use Committee under Experiment No. 93.0 and met the USDA and Federation of Animal Science Societies (FASS, 2010) guidelines for the care and use of animals. This study evaluated multigenerational crossbred commercial resource populations developed at USMARC over the past 2 decades. The initial Yorkshire, Landrace, and Duroc (YLD) population was established in 2001 and consisted of crossbred Yorkshire and Landrace populations of relative commercial genetics at their establishment in 1995 with an additional single sampling of maternal line sires of lean Landrace and terminal line sires of high marbling Duroc from multiple U.S. commercial genetic companies in 2001 (Lindholm-Perry et al., 2009). The YLD was maintained by *inter se* mating of 12 sire lines using batch farrowing system in 2-mo rotations with females only being retained through 2 parities for 10 yr. In 2011, continuous rotational sampling of commercially available maternal line purebred Landrace and Yorkshire genetic semen from multiple U.S. genetic companies (up to 6 companies sampled annually) was implemented initially utilizing founder YLD composite sows with weekly matings, each corresponding to individual farrowing groups and sows being retained through 4 parities (Wijesena et al., 2023). Subsequent matings have generated crossbred Landrace-sired or Yorkshire-sired populations that are greater than 50% of the corresponding genetics given that all sires are purebred for their respective breeds. Since 2011, annual rotation of breedings with purebred Landrace or Yorkshire sires to these crossbred dams has generated 2 unique crossbred populations.

This study evaluated matings of either purebred Yorkshire sires (*n* = 71) in 2018 or purebred Landrace sires (*n* = 73) in 2019 bred to crossbred populations of Landrace- (*n* = 357) or Yorkshire-sired dams (*n* = 448) across a 2-yr period. All matings were random between sire and dam across all parities within each farrowing group with a weekly rotation between commercial genetic companies (*n* = 3 for this sampling period) that provided purebred semen. As indicated above, dams were retained up to 4 parities, so a dam could be represented in multiple samplings up to fourth parity, or could have been culled, removed, or blood sampling missed, and only represented once with a range of sampling for individual dams from first to fourth parity. This generated 1,535 pregnancies representing mixed parity (1 to 4; average parity = 2.27) dams from various matings of purebred sires and crossbred dams across the 2 populations. The proportion of specific genetics for repeated or unique crossbred Landrace-sired and Yorkshire-sired dams utilized in this study are illustrated in Table 1. Table 2 illustrates the specific sample size for the genetics of dam line, sire line, and parity, and their corresponding dam line to sire line matings across parity represented in this study.

**Table 1.** Percentage of specific genetic background (Landrace, Yorkshire, or Duroc) for crossbred Landrace- and Yorkshire-sired dams utilized in this study to assess relationships of early plasma progesterone concentrations on subsequent farrowing litter statistics

Repeated dams				
Dam line	Sample size (*n*)	% Landrace	% Yorkshire	% Duroc
**Entire population**	1535	54.55 ± 0.67	44.76 ± 0.67	0.69 ± 0.01
** Landrace-sired**	830	75.77 ± 0.44	23.53 ± 0.44	0.70 ± 0.01
** Yorkshire-sired**	705	29.57 ± 0.45	69.74 ± 0.44	0.69 ± 0.01

Unique dams				
Dam line	Sample size (*n*)	% Landrace	% Yorkshire	% Duroc
**Entire population**	805	49.80 ± 0.92	49.50 ± 0.92	0.69 ± 0.02
** Landrace-sired**	357	75.48 ± 0.68	23.80 ± 0.68	0.72 ± 0.02
** Yorkshire-sired**	448	29.34 ± 0.56	69.99 ± 0.56	0.67 ± 0.02

Repeated dams (upper panel) correspond to dam observations across any parity (1 to 4) whereas unique dams (lower panel) correspond to single dam observations across multiple parities (1 to 4). Percentage data are presented as means ± SEM.

**Table 2.** Sample distribution for the number of litters evaluated across all 4 parities

Dam or sire line	Parity				Total by genotype
	1	2	3	4	
**Landrace-sired dams**	261	212	183	174	830
**Yorkshire-sired dams**	244	191	151	119	705
** Landrace sires**	237	195	166	179	777
** Yorkshire sires**	268	208	168	114	758
** Total by parity**	505	403	334	293	1535

Dam to sire line mating	Parity				Total by genotype
	1	2	3	4	
**Landrace-sired to Landrace**	6	65	132	159	362
**Landrace-sired to Yorkshire**	255	147	51	15	468
**Yorkshire-sired to Landrace**	231	130	34	20	415
**Yorkshire-sire to Yorkshire**	13	61	117	99	290
** Total by parity**	505	403	334	293	1535

The upper panel illustrates the sample size for dam or sire lines (Landrace or Yorkshire) across parities. The lower panel highlights the sample size for the respective matings between dams or sires of Landrace or Yorkshire genetics.

Following standard breeding protocols at USMARC, females were bred using conventional artificial insemination (AI) when first detected in standing estrus, designated as day 0 of pregnancy, and again 24 h later using the same single-sire semen within their corresponding weekly farrowing group. Single-sire semen was delivered on Tuesday for weekly farrowing groups, stored in 7-d extender, and AI was performed using sperm concentration of 2.5 to 3.0 × 10^9^ per insemination. Gilts (parity 0) were developed under standard management protocol (Wijesena et al., 2023) and were at least 210 d of age having at least one standing estrus prior to selection for breeding. Multiple parity dams (parities 1 to 3) were bred on their first estrus following weaning with an average weaning-to-estrus interval of 4.4 d for the dams sampled in this study. All females were bred and maintained in stalls for the first month of pregnancy and fed 2 kg/d of the standard USMARC gestation ration (Rempel et al., 2022) with ad libitum access to water. At approximately day 30 of pregnancy, ultrasound was used to confirm pregnancy. Pregnant females were moved to group gestation pens (1.6 m^2^/female; 52 females per pen/feeder) and fed 2.3 kg/d of the standard USMARC gestation ration (Rempel et al., 2022) via electronic sow feeding systems (Osborne Livestock Equipment, Osborne, KS) with *ad libitum* access to water through day 110 of pregnancy. At approximately day 110 of pregnancy, females were moved to individual farrowing stalls and fed 2.7 kg/d of the standard USMARC lactation ration (Rempel et al., 2022) with *ad libitum* access to water until farrowing.

### Blood sampling during early pregnancy and progesterone analysis

The standard breeding period for each weekly farrowing group at USMARC occurs Tuesday through Sunday with the majority (>90%) of dams being bred Tuesday through Friday within a given farrowing group. For this study, blood samples were collected at day 7 of pregnancy (day 0 = first day of breeding) the following week after breeding between Tuesday thru Friday to ensure maximum potential differences in P4 during the estrous cycle between dams as was previously demonstrated by Knox and colleagues (Knox et al., 2003). Within the 2-yr annual rotation of Yorkshire- and Landrace- sires, farrowing groups were continuously and randomly sampled with some weeks missing due to staff availability (ie, other experiments, building construction, holidays, and official travel) and a 35-d federal furlough in 2018 to 2019. This resulted in blood sampling for 44 farrowing groups from Yorkshire-sired litters and 50 farrowing groups from Landrace-sired litters.

To avoid stress for dams during early pregnancy, blood was collected using a previously described minimally invasive tail bleeding technique (Muirhead, 1981). Prior to blood sampling, a small amount of feed was provided to occupy the dam while taking the blood sample. Briefly, blood was collected from the coccygeal vein using a 3 cc syringe containing <0.2 mL of 4% EDTA and 21 gauge 38 mm needle. The skin on the underside of the tail was cleaned with 70% ethanol, the tail was held at an 80 to 90° angle, and the needle was inserted at a 45° angle approximately 13 mm toward the fifth coccygeal vertebra. This technique was useful for obtaining ∼1 to 2 mL of blood from dams, and no sample was collected if dams did not tolerate tail bleeding. Blood was transferred to a 2 mL cryovial, centrifuged at 468 × relative centrifugal force at 4 °C for 20 min to separate plasma. Volume of plasma was recorded for all samples (average = 440 µL/sample), and plasma was stored at −80 °C until assayed for P4.

Plasma P4 was measured using a commercial solid-phase radioimmunoassay (RIA; 0727010-CF; MP Biomedicals, Santa Ana, CA) with a detection sensitivity of P4 at 0.15 ng/mL according to the manufacturer’s protocol and previously validated for use in swine (Díaz et al., 2017). Each sample plasma was assayed for P4 in duplicate using 100 µL of plasma with an average intra-assay CV of 3.3%. Each assay (*n* = 10) had a predetermined high and low P4 reference control included to determine inter-assay CV (10.4% average CV across the 10 assays).

### Farrowing data: litter statistics, piglet weights, weight variation, and mortality

Approximately 12 to 24 h post-farrowing, standard USMARC day 1 litter processing was performed, and litter statistics were collected including counting the total number of pigs born (TB), which included the number born alive (NBA), and number born dead (NBD, including mummies and stillborn piglets). These numbers were used to calculate combined pre- and peri-natal piglet mortality (PNM; [NBD/TB] × 100) rate defined as the percentage of piglet death that occurred during late prenatal period (mummies), and shortly before or during farrowing (stillborn). Any live born piglets that died prior to day 1 processing were included in PWM calculations (see below). Sex was determined for all fully formed piglets (including born alive and stillborn) and these piglets were weighed to determine total and average litter BW. Within-litter BW variation was calculated for each litter using the CV of the average litter BW (BWCV). In addition, all live born piglets received ear tag identification during day 1 processing. To ensure adequate maternal care, cross-fostering was performed between day 1 and 2 of life where random piglets were moved from extremely large litters to smaller litters to normalize litter size when necessary. On approximately day 3 post-farrowing, all piglets were given iron supplements, tails were docked, and males were castrated. At approximately 4 wk post-farrowing, all piglets were weaned (average weaning age 26.5 d) from dams and weighed to determine total and average litter weaning weight (WW). Within-litter WW variation was calculated for each litter using the CV of the average litter WW (WWCV). To adjust for cross-fostering, PWM was calculated for individual littermates (Litter PWM) and lactating dams (Dam PWM). Litter PWM was calculated as (NBA—number piglets weaned in the litter/NBA × 100) and included piglets nursed directly by their dam as well as those cross-fostered to other dams. Dam PWM was calculated by adjusting NBA to account for piglets cross-fostered off and onto the dam as piglets nursed by dam (PND = NBA—piglets fostered off + piglets fostered on) and Dam PWM calculated as (PND—number piglets weaned by dam/PND × 100). During this study, another study was being conducted in a subset of eight farrowing groups (137 total litters) that randomly euthanized an average size healthy piglet during mid-lactation. Since these piglets did not have equal probability to be weaned, they were removed completely from the analysis for both Litter PWM and Dam PWM by removing them from NBA in these litters. Furthermore, twenty-three dams (14 Landrace and 9 Yorkshire dams) were removed from the analysis of Dam PWM that died or were euthanized during very early lactation and their entire litter of piglets was cross- fostered to other dams.

### Statistical analysis

Relationships between early gestation P4 levels in dams and corresponding farrowing data, piglet BW and WW, within-litter BW and WW variation, and PWM were evaluated by Pearson correlation analysis and means ± SEM for each variable were determined by PROC MEANS using SAS (Steel et al., 1997; SAS, 2003). In addition, the relationships between Dam PWM and corresponding farrowing data, piglet BW and WW, within-litter BW and WW variation, and Litter PWM were determined using Pearson correlation analysis. For simple correlation, Pearson correlation coefficients were determined for the entire population as well as the breakdown of Landrace or Yorkshire genetics within dam or sire lines, respectively.

To further determine the influence of dam or sire line between the Landrace or Yorkshire genetics, respectively, as well as dam parity on experimental variables, early gestation P4 levels, farrowing data, piglet BW and WW, within-litter BW and WW variation, and PWM were analyzed using PROC MIXED Model Procedures in SAS (Steel et al., 1997; SAS, 2003). The distribution of dams and sires represented across 4 parities was consistent across Landrace and Yorkshire genetics (Table 2). However, due to the annual rotation of Landrace and Yorkshire sires and management of gilts into the breeding system, dam by sire line matings had unequal parity distributions (Table 2). Most of the first and second parity dams were bred with semen from the opposite line (ie, Landrace to Yorkshire or Yorkshire to Landrace) due to the birth of gilt within 1 yr and corresponding development of gilt ages (first breeding ∼210 d) and breeding cycle (∼2 parities per year) in their second year of life bred to opposite line sires. In contrast, most of the third and fourth parity dams were intra-line matings (ie, Landrace to Landrace or Yorkshire to Yorkshire) bred in their third year of life to similar line sires. Given that parity is known to influence farrowing data (Wientjes et al., 2013; Sanz-Fernández et al., 2024), offset parity structure for dam by sire line made it difficult to interpret main effect interactions, particularly the three-way interaction between dam line by sire line by parity. As a result, multiple models were used to evaluate fixed main effects individually and interactions across fixed effects in a stepwise approach. Model 1 only included the fixed main effects of dam line, sire line, and parity. Model 2 included the fixed effects of dam line, sire line, and parity as well as the interactions between dam line by parity and sire line by parity. Model 3 included the fixed main effects of dam line, sire line and the one-way interaction between dam line by sire line. Given the unequal parity distribution across dam by sire line matings, Model 3 was analyzed with all parities combined. For all MIXED Model analyses, individual dams were analyzed as repeated effects within dam line and individual sires were analyzed as random effects within sire line with no covariates included in the models, and results are reported as least squares means ± SEM. When there was a significant *F*-statistic, mean differences were determined using the Dunnett multiple comparison test (Steel et al., 1997; SAS, 2003). Mean differences were considered significant differences when the *F*-statistic was *P *≤ 0.05 and tendencies when they were between *P *= 0.06 and *P *= 0.10.

## Results

### Relationship between P4 and farrowing data

Overall variable means and Pearson correlation coefficients are shown in Table 3 highlighting the relationship of early gestation P4 concentrations in pregnant dams and corresponding farrowing data for the entire population, as well as the breakdown of Landrace or Yorkshire genetics within dam or sire lines, respectively. These analyses demonstrate weak degrees of correlations (all *r* < ± 0.10 coefficients) between early gestation P4 levels and all corresponding farrowing data. When comparing data for the entire population, there was a significant positive correlation (*r* = 0.05379; *P *= 0.0352) between P4 and TB piglets. In contrast, there was a significant negative correlation (*r* = −0.05054; *P *= 0.0478) between P4 and piglet BW when evaluating the entire population. When comparing dam line genetics (Landrace or Yorkshire), only Yorkshire-sired dams had a significant negative correlation (*r* = −0.09872; *P *= 0.0087) between P4 and piglet BW. In contrast when comparing sire line genetics (Landrace or Yorkshire), Landrace-sired litters had significant positive correlations between P4 and TB, born dead, and stillborn piglets (*r* = 0.07622; *P *= 0.0337, *r* = 0.07029; *P *= 0.0502, and *r* = 0.07176; *P *= 0.0455, respectively), but a significant negative correlation between P4 and piglet BW (*r* = −0.0230; *P *= 0.0218). All other correlations between P4 and farrowing data were not significant, including piglet BWCV and PWM.

**Table 3.** Variable means (± SEM) and Pearson correlation coefficients for the relationship between early gestation progesterone (P4) on litter statistics, perinatal piglet mortality (PNM), average piglet birth and weaning weights, coefficient of variation for birth weight (BWCV) and weaning weight (WWCV), and preweaning piglet mortality of littermates (Litter PWM) and dams (Dam PWM) from dam lines and sire lines of either Landrace or Yorkshire genetics

Variable	Entire population	Dam lines		Sire lines	
		Landrace-sired	Yorkshire-sired	Landrace	Yorkshire
**P4 (ng/mL)**	13.97 ± 0.15	13.63 ± 0.20	14.37 ± 0.24	15.11 ± 0.20	12.81 ± 0.22
	1.00000	1.00000	1.00000	1.00000	1.00000
	*n* = 1535	*n* = 830	*n* = 705	*n* = 777	*n* = 758
**Total born (No.)**	13.89 ± 0.10	13.80 ± 0.13	14.00 ± 0.15	13.98 ± 0.14	13.80 ± 0.14
	0.05377	0.06131	0.04353	0.07622	0.02608
	***P = 0.0352***	*P = 0.0775*	*P = 0.2483*	***P = 0.0337***	*P = 0.4734*
**Born alive (No.)**	12.89 ± 0.09	12.80 ± 0.12	13.00 ± 0.14	12.95 ± 0.13	12.83 ± 0.14
	0.04034	0.03752	0.03986	0.05343	0.02384
	*P = 0.1141*	*P = 0.2802*	*P = 0.2905*	*P = 0.1368*	*P = 0.5122*
**Born dead (No.)**	1.00 ± 0.04	1.00 ± 0.06	0.99 ± 0.05	1.02 ± 0.05	0.97 ± 0.06
	0.03879	0.05810	0.01695	0.07029	0.00685
	*P = 0.1287*	*P = 0.0944*	*P = 0.6532*	***P = 0.0502***	*P = 0.8507*
**Mummies (No.)**	0.30 ± 0.02	0.31 ± 0.03	0.29 ± 0.02	0.25 ± 0.02	0.35 ± 0.03
	0.02306	0.03086	0.01476	0.01711	0.04935
	*P = 0.3666*	*P = 0.3746*	*P = 0.6956*	*P = 0.6340*	*P = 0.1747*
**Stillborn (No.)**	0.70 ± 0.03	0.70 ± 0.05	0.70 ± 0.05	0.77 ± 0.05	0.62 ± 0.05
	0.03262	0.05079	0.01191	0.07176	−0.02528
	*P = 0.2014*	*P = 0.1437*	*P = 0.7523*	***P = 0.0455***	*P = 0.4871*
**PNM (%)**	6.83 ± 0.27	0.70 ± 0.05	6.73 ± 0.37	6.88 ± 0.35	6.79 ± 0.42
	0.02284	0.05079	−0.00424	0.03749	0.01049
	*P = 0.3712*	*P = 0.1437*	*P = 0.9106*	*P = 0.2966*	*P = 0.7730*
**Birth weight (kg)**	1.44 ± 0.01	1.47 ± 0.01	1.41 ± 0.01	1.46 ± 0.01	1.44 ± 0.01
	−0.05054	0.00655	−0.09872	−0.08230	−0.02582
	***P = 0.0478***	*P = 0.8507*	***P = 0.0087***	***P = 0.0218***	*P = 0.4781*
**BWCV (%)**	19.44 ± 0.18	19.45 ± 0.25	19.42 ± 0.26	19.56 ± 0.25	19.30 ± 0.26
	0.02980	0.05372	0.00421	−0.01064	0.06156
	*P = 0.2439*	*P = 0.1227*	*P = 0.9112*	*P = 0.7675*	*P = 0.0908*
**Wean weight (kg)**	7.69 ± 0.03	7.80 ± 0.04	7.55 ± 0.04	7.78 ± 0.03	7.59 ± 0.04
	−0.010401	0.02417	−0.04046	−0.05828	−0.00893
	*P = 0.5838*	*P = 0.4873*	*P = 0.2837*	*P = 0.1048*	*P = 0.8063*
**WWCV (%)**	14.43 ± 0.14	14.43 ± 0.19	14.44 ± 0.20	14.18 ± 0.18	14.69 ± 0.20
	0.01091	0.05119	−0.03250	−0.00476	0.04254
	*P = 0.6705*	*P = 0.1428*	*P = 0.3900*	*P = 0.8949*	*P = 0.2449*
**Litter PWM (%)**	11.68 ± 0.31	11.24 ± 0.40	12.19 ± 0.48	11.21 ± 0.42	12.16 ± 0.45
	0.02078	0.05017	−0.01239	0.02695	0.03049
	*P = 0.4163*	*P = 0.1490*	*P = 0.7428*	*P = 0.4535*	*P = 0.4022*
**Dam PWM (%)**	12.26 ± 0.36	12.08 ± 0.49	12.47 ± 0.53	11.50 ± 0.46	13.04 ± 0.55
	0.01094	0.02881	−0.00923	0.04185	0.00573
	*P = 0.6707*	*P = 0.4112*	*P = 0.8079*	*P = 0.2467*	*P = 0.8760*

Bold *P*-values were different (*P* < 0.0502).

### Relationship between Dam PWM and farrowing data

Pearson correlation coefficients for the relationship between Dam PWM and other farrowing data are shown in Table 4. When evaluating the entire population, all the farrowing data illustrated significant correlations (*P *< 0.004) between Dam PWM, except PNM, which tended (*P *= 0.06) to correlate with Dam PWM. These correlations ranged from moderate to high degrees of correlation (ranging between *r* >−0.29314 and < 0.87987). Only piglet BW and WW demonstrated negative correlation with Dam PWM (*r* = −0.29314; *P *< 0.0001 and *r* = −0.07375; *P *= 0.0041, respectively) compared to positive correlations between the other farrowing variables including BWCV and WWCV (*r* = 0.27057; *P *< 0.0001 and *r* = 0.10230; *P *< 0.0001, respectively). As expected, Dam PWM was highly correlated with Litter PWM (*r* = 0.87987; *P *< 0.0001) for the entire population. When evaluating dam or sire line genetics (Landrace vs. Yorkshire), most of the relationships followed similar patterns and significance as was observed for the entire population (Table 4). However, the relationship between Dam PWM and PNM were not different (*P *> 0.11) among dam or sire line genetics, likely due to reduced sample size within these comparisons. In addition, the relationship of Dam PWM to number of mummies differed among the dam lines (*r* = 0.11309; *P *= 0.0012 and *r* = 0.04769; *P *= 0.2089) and sire lines (*r* = −0.02015; *P *= 0.5772 and *r* = 0.14946; *P *< 0.0001) genetics for Landrace and Yorkshire, respectively, but the relationship of Dam PWM to number of stillborn shifted between dam lines (*r* = 0.04030; *P *= 0.2502 and *r* = 0.13025; *P *= 0.0006) and sire lines (*r* = 0.15153; *P *< 0.0001 and *r* = 0.01401; *P *= 0.7028) genetics for Landrace and Yorkshire, respectively. There was also a significant negative correlation of Dam PWM to piglet WW for Landrace-sired dams compared to Yorkshire-sired dams (*r* = −0.09272; *P *= 0.0081 and *r* = −0.04823; *P *= 0.2041, respectively), but this negative correlation switched for Yorkshire sires compared to Landrace sires (*r* = −0.08600; *P *= 0.0190 and *r* = −0.04879; *P *= 0.1771, respectively). In addition, there was a greater degree for a positive correlation of Dam PWM and WWCV for Yorkshire-sired dams and sires (*r* = 0.15906; *P *< 0.0001 and *r* = 0.12134; *P *= 0.0009, respectively) compared to Landrace-sired dams and sires (*r* = 0.05073; *P *= 0.1489 and *r* = 0.07385; *P *= 0.0411, respectively). Furthermore, the degree of correlation between Dam PWM and farrowing traits varied by genetic line. For instance, Yorkshire-sired dams showed stronger positive correlations between Dam PWM and TB, NBA, and NBD. In contrast, Yorkshire sires had stronger negative correlations between Dam PWM and piglet BW and WW. These differences indicate that genetic line specifically affects the relationship between Dam PWM and farrowing traits in Landrace and Yorkshire dams and sires.

**Table 4.** Pearson correlation coefficients for the relationship between preweaning piglet mortality (Dam PWM) of dams on litter statistics, perinatal mortality (PNM), average piglet birth and weaning weights, coefficient of variation for birth weight (BWCV) and weaning weight (WWCV), and littermate PWM (Litter PWM) from dam lines and sire lines of either Landrace or Yorkshire genetics

Variable	Entire population	Dam line		Sire line	
		Landrace-sired	Yorkshire-sired	Landrace	Yorkshire
**Dam PWM (%)**	1.00000	1.00000	1.00000	1.00000	1.00000
	*n* = 1512	*n* = 816	*n* = 696	*n* = 768	*n* = 744
**Total born (No.)**	0.28209	0.21794	0.34915	0.31339	0.25848
	***P < 0.0001***	***P < 0.0001***	***P < 0.0001***	***P < 0.0001***	***P < 0.0001***
**Born alive (No.)**	0.25677	0.18954	0.32785	0.28614	0.23316
	***P < 0.0001***	***P < 0.0001***	***P < 0.0001***	***P < 0.0001***	***P < 0.0001***
**Born dead (No.)**	0.10743	0.09037	0.13122	0.12483	0.09713
	***P < 0.0001***	***P = 0.0098***	***P = 0.0005***	***P = 0.0005***	***P = 0.0080***
**Mummies (No.)**	0.08508	0.11309	0.04769	−0.02015	0.14946
	***P = 0.0009***	***P = 0.0012***	*P = 0.2089*	*P = 0.5772*	***P < 0.0001***
**Stillborn (No.)**	0.07846	0.04030	0.13025	0.15153	0.01401
	***P = 0.0023***	*P = 0.2502*	***P = 0.0006***	***P < 0.0001***	*P = 0.7028*
**PNM (%)**	0.04915	0.04909	0.04949	0.05706	0.04644
	***P = 0.0560***	*P = 0.1612*	*P = 0.1922*	*P = 0.1141*	*P = 0.2057*
**Birth weight (kg)**	−0.29314	−0.29679	−0.29130	−0.24410	−0.33663
	***P < 0.0001***	***P < 0.0001***	***P < 0.0001***	***P < 0.0001***	***P < 0.0001***
**BWCV (%)**	0.31358	0.29043	0.34148	0.30370	0.32678
	***P < 0.0001***	***P < 0.0001***	***P < 0.0001***	***P < 0.0001***	***P < 0.0001***
**Wean weight (kg)**	−0.07375	−0.09272	−0.04823	−0.04879	−0.08600
	***P = 0.0041***	***P = 0.0081***	*P = 0.2041*	*P = 0.1771*	***P = 0.0190***
**WWCV (%)**	0.10114	0.05073	0.15906	0.07385	0.12134
	***P < 0.0001***	*P = 0.1489*	***P < 0.0001***	***P = 0.0411***	***P = 0.0009***
**Litter PWM (%)**	0.87987	0.85861	0.90402	0.90350	0.86113
	***P < 0.0001***	***P < 0.0001***	***P < 0.0001***	***P < 0.0001***	***P < 0.0001***

Bold *P*-values were different (*P* < 0.0560).

### Dam line, sire line, and parity main effects on P4 and farrowing data (Model 1)

To further assess the influence of genetic line and parity on early gestation P4 and farrowing variables in this population, a MIXED model analysis was used to first identify the fixed main effects of dam line, sire line, and parity (Model 1) on these variables and is presented in Table 5. When comparing dam line genetics, TB and NBA tended (*P *< 0.07) to be greater in litters from Yorkshire-sired dams compared to Landrace-sired dams. Piglet BW, WW, and Litter PWM were different (*P *< 0.05) between Landrace-sired and Yorkshire-sired dams, where BW & WW were greater in litters from Landrace-sired dams compared to Yorkshire-sired dams, but Litter PWM was greater for piglets from Yorkshire-sired dams. When comparing sire line genetics (Table 5), early gestation P4 was greater (*P *< 0.0001) in litters sired by Landrace boars compared to Yorkshire boars. The number of mummies was greater (*P *= 0.0141) in litters sired by Yorkshire boars, but conversely the number of stillborn tended (*P *= 0.0705) to be greater in litters sired by Landrace boars. Weaning weight was greater (*P *= 0.0008) for Landrace-sired litters, but WWCV was greater (*P *= 0.0079) for Yorkshire-sired litters. Litter PWM and Dam PWM were both greater (*P* < 0.06) in litters sired by Yorkshire boars compared to Landrace boars (Table 5).

**Table 5.** Least squares means ± SEM and *P-*values following ANOVA using MIXED model analysis (Model 1) for the fixed main effects of dam line, sire line, and parity on early gestational progesterone (P4), litter statistics, perinatal piglet mortality (PNM), average piglet birth and weaning weights, coefficient of variation for birth weight (BWCV) and weaning weight (WWCV), and preweaning piglet mortality of littermates (Litter PWM) and dams (Dam PWM)

Variable	Dam Line		Sire Line		Parity			
	Landrace-sired	Yorkshire-sired	Landrace	Yorkshire	1	2	3	4
**P4 (ng/mL)**	13.7 ± 0.3	14.2 ± 0.3	14.9 ± 0.3	12.9 ± 0.3	14.4 ± 0.3^a^	13.1 ± 0.3^b^	13.7 ± 0.4^a,b^	14.6 ± 0.4^a^
	*P = 0.1113*		***P < 0.0001***		***P = 0.0016***			
**Total born (no.)**	14.0 ± 0.1	14.3 ± 0.2	14.1 ± 0.2	14.2 ± 0.2	12.1 ± 0.2^a^	13.9 ± 0.2^b^	15.2 ± 0.2^c^	15.5 ± 0.2^c^
	*P = 0.0705*		*P = 0.8123*		***P < 0.0001***			
**Born alive (no.)**	13.0 ± 0.1	13.3 ± 0.1	13.1 ± 0.1	13.2 ± 0.1	11.2 ± 0.2^a^	13.0 ± 0.2^b^	14.1 ± 0.2^c^	14.2 ± 0.2^c^
	*P = 0.0608*		*P = 0.6734*		***P < 0.0001***			
**Born dead (no.)**	1.03 ± 0.06	1.03 ± 0.06	1.04 ± 0.06	1.02 ± 0.2	0.87 ± 0.07^a,b^	0.84 ± 0.08^a^	1.06 ± 0.08^b^	1.34 ± 0.09^c^
	*P = 0.9633*		*P = 0.7994*		***P < 0.0001***			
**Mummies (no.)**	0.31 ± 0.03	0.31 ± 0.03	0.27 ± 0.03	0.36 ± 0.03	0.26 ± 0.03	0.31 ± 0.04	0.32 ± 0.4	0.32 ± 0.04
	*P = 0.8731*		***P = 0.0141***		*P = 0.3627*			
**Stillborn (no.)**	0.72 ± 0.05	0.72 ± 0.05	0.78 ± 0.05	0.65 ± 0.05	0.62 ± 0.06^a,c^	0.53 ± 0.06^a^	0.74 ± 0.07^c^	0.99 ± 0.08^d^
	*P = 0.9690*		*P = 0.0705*		***P < 0.0001***			
**PNM (%)**	6.96 ± 0.39	6.81 ± 0.42	6.90 ± 0.41	6.87 ± 0.42	7.11 ± 0.49^a^	5.78 ± 0.54^b^	6.43 ± 0.59^c^	8.22 ± 0.64^c^
	*P = 0.7980*		*P = 0.9665*		***P < 0.0228***			
**Birth weight (kg)**	1.48 ± 0.01	1.41 ± 0.02	1.45 ± 0.01	1.44 ± 0.01	1.43 ± 0.01^a^	1.48 ± 0.01^b^	1.44 ± 0.01^a^	1.42 ± 0.01^a^
	***P < 0.0001***		*P = 0.3766*		***P < 0.0010***			
**BWCV (%)**	19.9 ± 0.2	20.1 ± 0.3	19.9 ± 0.3	20.1 ± 0.27	15.5 ± 0.3^a^	19.5 ± 0.3^b^	21.8 ± 0.4^c^	23.2 ± 0.4^d^
	*P = 0.4087*		*P = 0.8123*		***P < 0.0001***			
**Weaning weight (kg)**	7.83 ± 0.04	7.67 ± 0.04	7.82 ± 0.05	7.59 ± 0.05	7.39 ± 0.05^a^	7.85 ± 0.05^b,c^	7.89 ± 0.06^b^	7.70 ± 0.06^c^
	***P < 0.0001***		***P = 0.0008***		***P < 0.0001***			
**WWCV (%)**	14.6 ± 0.2	14.9 ± 0.2	14.4 ± 0.2	15.1 ± 0.2	12.5 ± 0.2^a^	14.5 ± 0.2^b^	15.4 ± 0.3^c^	16.5 ± 0.3^d^
	*P = 0.2471*		***P = 0.0079***		***P < 0.0001***			
**Litter PWM (%)**	11.3 ± 0.4	12.8 ± 0.5	11.4 ± 0.5	12.7 ± 0.5	9.7 ± 0.6^a^	10.0 ± 0.6^a^	14.1 ± 0.7^b^	14.4 ± 0.7^b^
	***P = 0.0218***		***P = 0.0575***		***P < 0.0001***			
**Dam PWM (%)**	12.1 ± 0.5	13.1 ± 0.6	11.7 ± 0.6	13.5 ± 0.6	10.2 ± 0.7^a^	10.4 ± 0.7^a^	14.4 ± 0.8^b^	15.4 ± 0.8^b^
	*P = 0.1791*		***P = 0.0321***		***P < 0.0001***			

Columns with different superscripts were different (*P *< 0.0228) across parity. Bold *P*-values were different (*P* < 0.0575).

When comparing fixed effect of parity (Table 5), all measured variables, except number of mummies, differed (*P *< 0.02) across one of the 4 parities. Early gestation P4 concentration was greatest (*P *= 0.0016) in first and fourth parity dams with a decrease in P4 concentration observed in second parity dams followed by a corresponding increase in third and fourth parity dams. Total born and NBA increased (*P *< 0.0001) as dams moved from first, second, and third parity but leveled off by fourth parity such that no differences in TB or NBA were observed between third and fourth parity. Number born dead, stillborn numbers, and PNM increased (*P *< 0.02) as dams transitioned between the 4 parities with the greatest number of these losses occurring in the fourth parity. Piglet BW was greatest (*P *< 0.0010) in piglets from second parity dams compared to other parities, but no differences in BW were observed from first, third, or fourth parity dams. However, the variation in piglet BW (BWCV) increased (*P *< 0.0001) progressively as dams transition through 4 parities. Similarly, piglet WW was greatest in litters from second and third parity dams compared to first parity dams but decreased in third to fourth parity. Within-litter variation (BWCV and WWCV) increased (*P *< 0.0001) progressively as parity advanced. Finally, both Litter and Dam PWM were greatest (*P *< 0.0001) in third and fourth parity dams compared to first and second resulting in >4% increase in mortality rates between early (first and second) and later (third and fourth) parities, respectively.

### Dam by parity and sire by parity interactions on P4 and farrowing data (Model 2)

**Figure 1.**
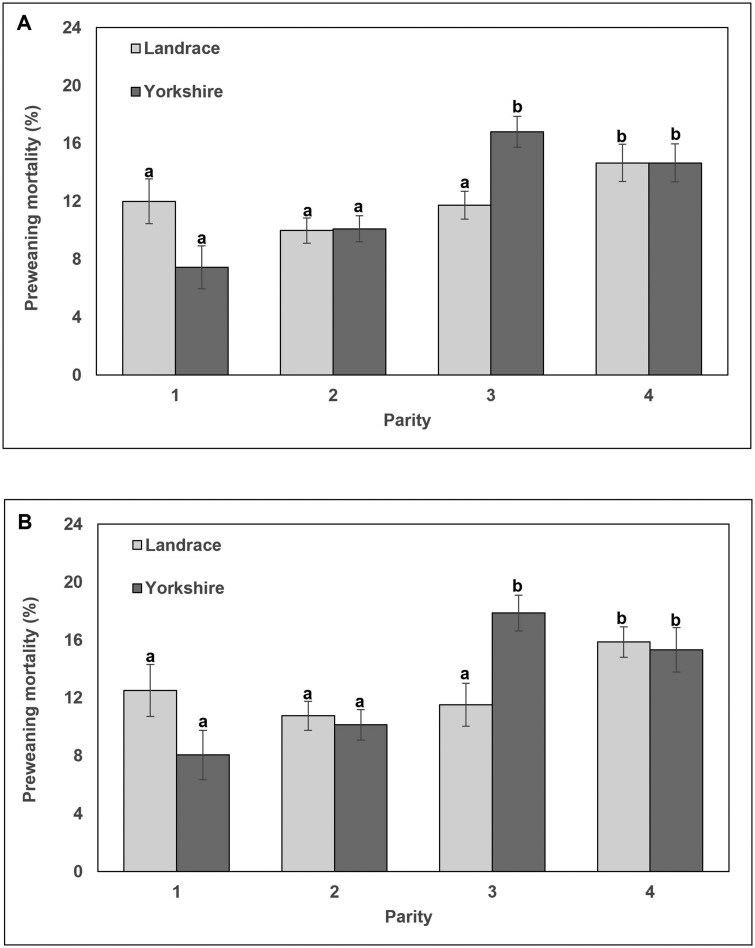
Interactions of dam genetics (Landrace, light grey bars or Yorkshire, dark grey bars) by parity (1 to 4) on litter preweaning mortality rate (A) and dam preweaning mortality rate (B). Values are reported as least squares means ± SEM as determined by MIXED model analysis for ANOVA. Columns with different superscripts were different (*P *< 0.0034) across the interaction (dam by parity).

**Figure 2.**
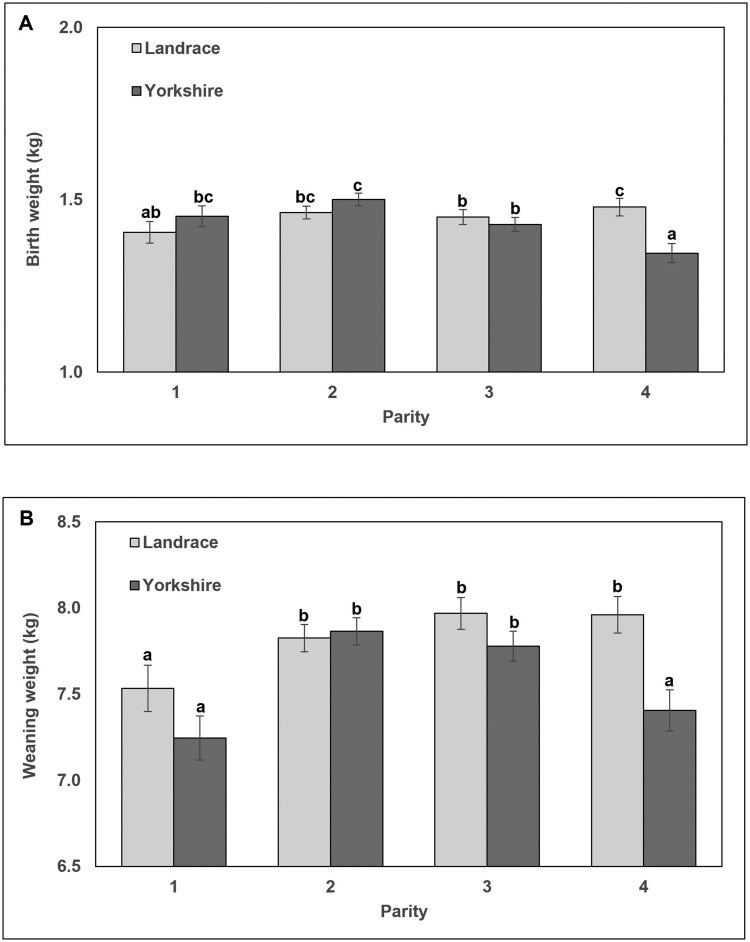
Interactions of sire genetics (Landrace, light grey bars or Yorkshire, dark grey bars) by parity (1 to 4) on piglet birth weight (A) and weaning weight (B). Values are reported as least squares means ± SEM as determined by MIXED model analysis for ANOVA. Columns with different superscripts were different (*P *< 0.0403) across the interaction (sire by parity).

Next, a MIXED Model analysis was used to evaluate two-way interactions of dam or sire genetics by parity (Model 2; Table 6). When analyzing dam line by parity interaction (Table 6), only litter and dam PWM had significant (*P *< 0.0037) interactions across these variables. Figure 1 illustrates the dam by parity interactions for Litter and Dam PWM, which did not differ between Landrace- and Yorkshire-sired dams in their first or second parity as well as in their fourth parity although PWM was greater in fourth parity dams compared to first and second parity dams, regardless of dam genetics. The interaction for both Litter (Figure 1A) and Dam (Figure 1B) PWM was observed in third parity dams where Yorkshire-sired dams had the greatest PWM rates (16.8 ± 1.0% and 17.9 ± 1.5% for Litter and Dam PWM, respectively) among all other parities and dam genetics. Interestingly, third parity Landrace-sired dams had similar PWM rates with earlier parity dams (11.7 ± 1.0% and 11.5 ± 1.1% for Litter and Dam PWM, respectively), illustrating better survivability in these litters. When analyzing the two-way interactions of sire line by parity (Table 6), only piglet BW and WW had significant (*P* < 0.04) interactions across these variables. Figure 2 demonstrates the sire by parity interactions for piglet BW and WW. Piglet BW (Figure 2A) and WW (Figure 2B) were not different in Landrace- or Yorkshire-sired litters during parity 1, 2, or 3. However by fourth parity, both piglet BW and WW were reduced in Yorkshire-sired litters compared to Landrace-sired litters and these BW and WW from fourth parity Yorkshire-sired litters were similar to parity 1 litters.

**Table 6.** *P-*values following ANOVA using MIXED model analysis (Model 2) for the fixed effects of dam line, sire line, parity, interaction of dam line by parity, and interaction of sire line by parity on early gestational progesterone (P4), litter statistics, perinatal piglet mortality (PNM), average piglet birth and weaning weights, coefficient of variation for birth weight (BWCV) and weaning weight (WWCV), and preweaning piglet mortality of littermates (Litter PWM) and dams (Dam PWM)

Variable	*Pr > F* for fixed effects				
	Dam line	Sire line	Parity	Dam * Parity	Sire * Parity
**P4 (ng/mL)**	*0.8679*	*0.0048*	*0.0014*	*0.3267*	*0.4977*
**Total born (No.)**	*0.9502*	*0.8345*	*<0.0001*	*0.0625*	*0.1457*
**Born alive (No.)**	*0.7396*	*0.8207*	*<0.0001*	*0.1649*	*0.0830*
**Born dead (No.)**	*0.5515*	*0.8569*	*<0.0001*	*0.5806*	*0.8614*
**Mummies (No.)**	*0.4736*	*0.0070*	*0.4095*	*0.1654*	*0.7947*
**Stillborn (No.)**	*0.7608*	*0.1575*	*<0.0001*	*0.5451*	*0.9581*
**PNM (%)**	*0.7217*	*0.7259*	*0.0462*	*0.7793*	*0.9015*
**Birth weight (kg)**	*0.1800*	*0.4308*	*0.0007*	*0.1920*	***0.0051***
**BWCV (%)**	*0.6093*	*0.1867*	*<0.0001*	*0.2145*	*0.3588*
**Wean weight (kg)**	*0.0144*	*0.0106*	*<0.0001*	*0.3085*	***0.0403***
**WWCV (%)**	*0.4271*	*0.0152*	*<0.0001*	*0.6387*	*0.7134*
**Litter PWM (%)**	*0.8771*	*0.8047*	*<0.0001*	***0.0081***	*0.0611*
**Dam PWM (%)**	*0.8797*	*0.9824*	*<0.0001*	***0.0034***	*0.1389*

Bold *P*-values were different (*P* < 0.0403) for interactions.

### Dam line by sire line interactions on P4 and farrowing data (Model 3)

Finally, to identify influences of matings between dam and sire genetics on early gestation P4 and corresponding farrowing data, a MIXED Model analysis was used to evaluate two-way interactions for dam to sire line matings (Model 3; Table 7). To consider the offset sample size of parities in these matings (see above), these analyses were performed by combining all parities (1 to 4) together. Most of these variables had significant (*P *< 0.02) dam line by sire line interactions when combining all parities together, except for number of mummies, PNM, and piglet BW (Table 7). Again, early gestation P4 concentrations were greater (*P *= 0.0259) in Landrace-sired litters compared to Yorkshire-sired litters regardless of dam genetics. Interestingly, P4 concentrations were greater (*P* = 0.0259) from Yorkshire-sired dams bred with Landrace boars compared to the Landrace intra-line matings. Weaning weight was greatest (*P *= 0.0126) in piglets from Landrace to Landrace matings compared to all other matings (Landrace to Yorkshire, Yorkshire to Landrace, and Yorkshire to Yorkshire; Table 7). Total born, NBA, NBD, stillborns, and BWCV were greater (*P *< 0.0001) in litters from intra-line matings (Landrace to Landrace or Yorkshire to Yorkshire) compared with reciprocal matings (Landrace to Yorkshire or Yorkshire to Landrace). Finally, WWCV, Litter PWM, and Dam PWM were greatest (*P *= 0.0001) in litters from intra-line matings between Yorkshire-sired dams and sires, followed by intra-line matings between Landrace-sired dams and sires, and were lowest in litters from reciprocal matings. Dam PWM from reciprocal Landrace-sired dam to Yorkshire sire matings was intermediate between intra-line Landrace and reciprocal Yorkshire-sired dam by Landrace sire matings.

**Table 7.** *P-*values following ANOVA using MIXED model analysis (Model 3) with all parities combined for the fixed effects of dam line, sire line, and interaction of dam line by sire line on early gestational progesterone (P4), litter statistics, perinatal piglet mortality (PNM), average piglet birth and weaning weights, coefficient of variation for birth weight (BWCV) and weaning weight (WWCV), and preweaning piglet mortality of littermates (Litter PWM) and dams (Dam PWM) from crosses between Landrace (L) and Yorkshire (Y) genetics

	*Pr > F*			Dam line to Sire line Interaction			
Variable	Dam	Sire	D*S	L to L	L to Y	Y to L	Y to Y
**P4 (ng/mL)**	*0.1608*	*<0.0001*	***0.0259***	14.40 ± 0.36^a^	12.97 ± 0.34^b^	15.51 ± 0.35^c^	12.71 ± 0.39^b^
**Total born (No.)**	*0.2072*	*0.9978*	***<0.0001***	14.87 ± 0.21^a^	12.93 ± 0.19^b^	13.17 ± 0.20^b^	15.12 ± 0.23^a^
**Born alive (No.)**	*0.1645*	*0.8356*	***<0.0001***	13.68 ± 0.20^a^	12.09 ± 0.18^b^	12.30 ± 0.19^b^	13.98 ± 0.22^a^
**NBD (No.)**	*0.8789*	*0.6708*	***0.0001***	1.19 ± 0.08^a^	0.85 ± 0.07^b^	0.88 ± 0.08^b^	1.15 ± 0.09^a^
**Mummies (No.)**	*0.9235*	*0.0155*	*0.2344*	0.27 ± 0.04	0.33 ± 0.04	0.23 ± 0.04	0.38 ± 0.05
**Stillborn (No.)**	*0.8096*	*0.0476*	***<0.0001***	0.92 ± 0.07^a^	0.52 ± 0.06^b^	0.64 ± 0.07^b,c^	0.76 ± 0.08^a,c^
**PNM (%)**	*0.7195*	*0.8542*	*0.1991*	7.38 ± 0.58	6.56 ± 0.51	6.47 ± 0.54	7.08 ± 0.64
**Birth weight (kg)**	*<0.0001*	*0.4341*	*0.1535*	1.47 ± 0.01	1.48 ± 0.01	1.42 ± 0.01	1.39 ± 0.02
**BWCV (%)**	*0.8857*	*0.9956*	***<0.0001***	21.50 ± 0.38^a^	17.80 ± 0.34^b^	17.85 ± 0.36^b^	21.55 ± 0.42^a^
**Wean weight (kg)**	*<0.0001*	*0.0011*	***0.0126***	8.00 ± 0.06^a^	7.64 ± 0.06^b^	7.59 ± 0.06^b^	7.51 ± 0.07^b^
**WWCV (%)**	*0.5223*	*0.0208*	***<0.0001***	15.15 ± 0.28^a^	13.83 ± 0.25^b^	13.34 ± 0.26^b^	15.99 ± 0.31^c^
**Litter PWM (%)**	*0.0542*	*0.1572*	***<0.0001***	12.21 ± 0.67^a^	10.05 ± 0.61^b^	10.24 ± 0.64^b^	14.41 ± 0.74^c^
**Dam PWM (%)**	*0.5282*	*0.0390*	***0.0001***	13.55 ± 0.91^a^	12.24 ± 0.82^a,b^	10.89 ± 0.86^b^	16.06 ± 1.00^c^

Least squares means ± SEM for variables are reported for the dam line by sire line interaction. Bold *P*-values were different (*P* < 0.0259) for interactions. Columns with different superscripts were different (*P *< 0.0259) across the interaction (dam line to sire line).

## Discussion

As litter size has increased considerably in commercial U.S. swine production over the past 2 decades, there has been a consequential negative affect on piglet BW, resulting in increased within-litter BW variation and greater PWM (Miles et al., 2023). The present study aimed to clarify whether natural variation in maternal plasma P4 concentrations during early gestation are associated with litter performance traits (e.g., NBA, piglet BW and WW, within-litter BW and WW variation, and PWM) in crossbred Landrace-sired and Yorkshire-sired dams bred to purebred sires from these breeds and evaluated potential genetic line and parity influences on these traits. To our knowledge, this is the first large-scale evaluation reporting the relationship of early gestation P4 on subsequent litter traits in commercial U.S. pig breeds. While there was a small (*r* < ±0.10) but significant positive correlation observed between P4 and TB piglets and a negative correlation between P4 and piglet BW, there were no significant direct correlations between P4 and within-litter BW variation or PWM when evaluating the entire dataset, contrary to our initial hypothesis. Despite the well-established biological importance of P4 for early pregnancy establishment, embryonic and fetal development (Geisert and Schmitt, 2002), our findings show only modest and variable relationships between early gestational P4 concentrations and key farrowing outcomes in commercial U.S. swine herds.

Exogenous P4 supplementation experiments to increase P4 during early gestation have had mixed results in improving reproductive outcomes in swine, particularly focusing on early embryonic development, survival, litter size, and farrowing success (Tyree and Stenhouse, 2025). Some of these studies, particularly when P4 is supplemented early in the estrous cycle (<day 3), have reported negative effects on pregnancy rate, embryonic survival and development, and litter size primarily due to altering the uterine synchrony (Mao and Foxcroft, 1998; Vallet and Christenson, 2004; Soede et al., 2012). Other studies have demonstrated positive pregnancy outcomes from exogenous P4 supplementation in embryonic survival (Jindal et al., 1997), litter size and placental function (Ashworth, 1991), and potential improved uterine function (Vallet et al., 1998; Szymanska and Blitek, 2016). A recent series of studies from Brazil targeting day 6 to 12 of gestation P4 supplementation, in range with the current study’s evaluation of natural P4 concentrations, demonstrated P4 supplementation improved uterine function and embryonic development in sows, but not gilts (Muro et al., 2020). These authors also reported increased litter size and live births with decreased number of small piglets (<800 g) and marginally decreased within-litter variation of BW in sows supplemented P4 during this period, suggesting that increased P4 could improve litter uniformity and piglet BW in sows (Muro et al., 2022).

The current study does show a slight positive correlation of early pregnancy P4 and TB piglets in the total dataset, likely due to the indirect selection for increased OR as litter size has increased (Da Silva et al., 2018) and resulting in greater P4 as OR increases as previously highlighted by Knox and colleagues (Knox et al., 2003). There was a consequential negative correlation of P4 on piglet BW, again likely due to increased OR resulting in uterine crowding and larger litter size resulting in reduced BW (Vallet, 2000); however, there were no direct relationships of early gestation P4 on within-litter BW variation or PWM. This suggests that naturally occurring variation in P4 during early gestation is not sufficient to induce an effect on uterine and embryonic development such that exogenous treatments can induce and thus may not have a direct influence on within-litter variation and piglet survival. However, litter size was increased, and piglet BW was reduced with elevated early gestational P4, suggesting some indirect effects on within-litter variation and piglet survival through increased litter size and decreased piglet BW. Furthermore, there are differences in P4 when accounting for dam and sire genetics suggesting that the genetic background of both the dam and sire may modulate the extent to which early gestational P4 supports conceptus development, piglet BW, and ultimately affects pre- and neo-natal survival.

When evaluating the dam and sire genetic lines relationship of early gestation P4 on these litter traits, Yorkshire-sired dams had a more pronounced negative relationship between P4 and piglet BW compared to Landrace-sired dams and this trend held up in all the analyses with Yorkshire-sired dams having reduced piglet BW compared to Landrace-sired dams. This suggests that increased P4 and corresponding decreased piglet BW in Yorkshire-sired dams may reflect greater OR, since P4 increases as OR increases (Knox et al., 2003), resulting in greater fetal crowding and decreased BW (Vallet, 2000). We have been evaluating OR at slaughter in these lines over the past 10 yr at the USMARC and an analysis of OR from 2955 females of equal distribution from these dam lines adjusted for age of female illustrates that this population of Yorkshire-sired females have greater (*P *< 0.0001) OR compared to the Landrace-sired females (20.0 ± 0.1 vs. 19.2 ± 0.1, respectively, for Yorkshire vs. Landrace; Miles et al. unpublished). There is limited literature reporting direct comparison of reproductive traits, particularly OR, and other key litter statistics in commercial U.S. Landrace and Yorkshire populations, partially due to the proprietary nature of swine production in the U.S. making it difficult to compare our data to other published studies on similar U.S. breeds. Data from Thailand using populations of purebred, European Landrace and Yorkshire genetics illustrated increased OR from Yorkshire gilts compared to Landrace gilts (Tantasuparuk et al., 2005), supporting the OR differences observed at USMARC. Although OR was not determined in dams of the current study, the OR differences among these populations support the hypothesis that increased OR in Yorkshire-sired dams resulted in the elevated P4 and the corresponding negative relationship on piglet BW as observed in the current study. An evaluation of OR and corresponding P4 levels at various stages of pregnancy and corresponding embryonic and fetal development in these dam lines are currently ongoing to clarify this hypothesis. Furthermore, Yorkshire-sired dams tended to have larger litters with significant reduction in BW and WW, and this resulted in greater litter PWM (particularly, in the third parity, Figure 1) compared to Landrace-sired dams. This potentially highlights an indirect negative relationship of PWM and P4 as increased P4 in Yorkshire-sired dams is associated with decreased BW and this decreased BW could contribute to increased PWM from these dams.

On the sire genetic background, there were significant positive relationships between early gestational P4 and TB, NBD, and stillborn piglets and a negative relationship in P4 and piglet BW in Landrace-sired litters. Interestingly, when comparing early gestation P4 concentration using ANOVA across the dam and sire line genetics, only Landrace-sired litters regardless of the genetics of the dam had greater P4 concentrations compared to Yorkshire-sired litters, consistent with the observed Pearson correlation relationship. This finding was quite unexpected given that this population of Yorkshire-sired dams has greater OR compared to Landrace-sired dams (see above), which likely resulted in a slight increase in litter size in Yorkshire-sired dams (+0.3 TB) with only a numerical increase in P4 (+0.5 ng/mL) in Yorkshire-sired dams compared to Landrace-sired dams in the current study. However, there is a significant increase in P4 in Landrace-sired litters (+2.0 ng/mL greater compared to Yorkshire-sired litters) regardless of dam illustrating a potential sire influence on early gestational P4 concentrations. There is growing evidence that seminal fluids during mating directly influence ovarian function in a variety of mammalian species (Robertson, 2007; Schjenken and Robertson, 2020). In the pig, studies have shown that seminal plasma advances ovulation timing (Waberski et al., 1997), elicits immune response in the oviduct and uterus (O’Leary et al., 2004; Bai et al., 2018), and increases P4 production in the follicular fluid during estrus and serum plasma during the estrous cycle in the absence of altered OR or CL weight (O’Leary et al., 2006). Growth factors, hormones, and recently extracellular vesicles have been proposed as the driving factors within seminal fluids that result in altered ovarian function (Madej et al., 2005; Bai et al., 2018; Barranco et al., 2024). Therefore, it is possible that Landrace seminal fluids may have a different molecular makeup compared to Yorkshire seminal fluids that could be driving the 15% increase in P4 regardless of genetics of the dam observed in the current study. Interestingly, Landrace-sired litters had greater BW and WW, particularly in the fourth parity (Figure 2), and this resulted in decreased PWM compared to Yorkshire-sired litters, irrespective of dam parity. This illustrates a potential indirect positive relationship of increased early gestational P4 in Landrace-sired litters by contributing to increased BW, WW and improved preweaning survival compared to Yorkshire-sired litters and merits further investigation.

More consistent and biologically relevant relationships were observed between PWM and key farrowing traits in the current study. As expected, Dam PWM was highly correlated (*r* = 0.88) with litter PWM confirming the reliability of these measures and reflecting how sow-level factors translate into litter-level outcomes. Our study showed that as litter size increased there was a positive relationship to increased PWM, and this was supported across many recent studies utilizing a variety of swine genetics (Knol et al., 2022; Knap et al., 2023; Sanz-Fernández et al., 2024). In addition, higher within-litter BW variation was positively associated with greater PWM, while higher mean BW was protective against piglet loss, which is consistent with previous reports (van der Lende et al., 2001; Damgaard et al., 2003; Knap et al., 2023; Harper and Bunter, 2024). As highlighted above, there were also unique responses from both dam and sire genetics in these farrowing data that contributed to differences in the Landrace and Yorkshire breeds to these key litter traits and corresponding PWM. These relationships and litter trait patterns illustrate a general trend for increased piglet BW and improved preweaning survival for Landrace compared to Yorkshire genetics. Previous literature from Europe has demonstrated that Landrace typically performed better than Yorkshire in terms of piglet BW and preweaning survival (Varona et al., 2007; Nielsen et al., 2013; Nowak et al., 2020), further supporting the current study observations. The correlations of PWM and corresponding litter traits reinforce that management strategies aiming to improve uniformity of fetal growth and increased piglet BW remain essential for mitigating piglet losses prior to weaning, regardless of natural variations in maternal P4 during early gestation.

The influence of dam parity on early gestation P4 levels, litter traits, and PWM was prominent in the current study. Early gestational P4 was greatest in the first and fourth parity dams with a significant decrease during the second parity followed by a rebounding trend in the third and fourth parities. Given that OR increases as dams advance through multiple parities (Da Silva et al., 2016) and early gestation P4 increases with increased OR (Knox et al., 2003), greater P4 was expected in the later parity dams. Interestingly, early gestation P4 was increased in first parity dams similar to fourth parity dams, which was unexpected given first parity dams have lower OR than later parity dams (Da Silva et al., 2016; Da Silva et al., 2017). Similar results have been observed in Brazil where gilts had greater concentration P4 per CL number compared to multiparous sows (Muro et al., 2021). Although the exact reasons for elevated P4 in first parity dams in the current study are not clear, one explanation could be due to the change in feed intake in diets of gilts entering their first parity breeding. In our system, gilts are moved into gilt development at approximately 150 d of age and fed 3 kg/d of gilt development ration (Rempel et al., 2022; Wijesena et al., 2023). Once gilts are selected for breeding, they are moved into stalls for breeding and immediately transitioned onto a gestation ration and feed is reduced to 2 kg/d (Rempel et al., 2022) throughout breeding and the early stages of pregnancy. Previous research has illustrated that increased feed intake (flush feeding; 25% increase) in gilts resulted in a significant reduction in P4 within 72 h following breeding (Jindal et al., 1997) and this decreased P4 level is due to increased metabolic clearance rate (MCR) of P4 in elevated feed intake gilts (Miller et al., 1999). Therefore, increased P4 level in first parity gilts observed in the current study likely resulted from the 33.3% decrease in feed intake (3 kg- to 2 kg-/d) that occurs at the time of the first breeding and thus reducing the MCR of P4, thereby, increasing circulating P4.

As expected, litter size variables (e.g., TB, NBA, NBD and stillborns) were highly influenced by dam parity in the current study with these variables greatest in the third and fourth parity dams. Increased litter size as dams advance through multiple parities has been consistently observed in swine herds of diverse genetics and environmental conditions throughout the world (Wientjes et al., 2013; Gourley et al., 2020; Ju et al., 2021; Buthelezi et al., 2024; Sanz-Fernández et al., 2024) with increased litter size in later parities likely driven by increased OR as dams advance through parities (Da Silva et al., 2016). The influence of parity on piglet BW in the current study was not as apparent as litter size as the largest piglets were born from second parity dams, but no differences were observed in first, third and fourth parity dams. This observation is consistent with previous evaluations that illustrate piglet BW is greatest in mid-parity dams (second and third) compared to first parity and later (fourth parity+) dams (Lavery et al., 2019; Buthelezi et al., 2024). Interestingly, within-litter variation (BWCV & WWCV) increased progressively as dams advanced through parity 4 in the current study with first parity dams having less variation compared to later parity dams regardless of limited differences in piglet BW, consistent with the observations of these studies (Lavery et al., 2019; Buthelezi et al., 2024). In the current study, first parity dams had substantially reduced litter size (<3.0 TB or NBA), but similar piglet BW with decreased (improved) within-litter BW variation (<6% BWCV) compared to third and fourth parity dams. These findings support previous observations that young parity dams are less sensitive to greater within-litter BW variation due to reduced litter size compared to multiparous dams (Quesnel et al., 2008). However, piglet BW was reduced in first parity dams in the current study, likely due to the fact that these dams are still growing during their first parity (Rempel et al., 2015) and thus may not provide the optimal uterine environment for fetal growth for the entire litter.

The current study found that third and fourth parity dams had greater PWM rates (>4.0%) compared to first and second parity and these findings are consistent with previous reports that increased litter size, reduced piglet BW, and greater within-litter BW variation led to greater PWM (van der Lende et al., 2001; Damgaard et al., 2003; Kemp et al., 2018). Again as OR increase in later parity dams, this resulted in increased litter size with increased fetal crowding that affected fetal growth and development (Vallet, 2000) and corresponded to increase within-litter BW variation and PWM (Da Silva et al., 2016; Da Silva et al., 2018). Taken together these results highlight the cumulative effects of advancing parity on uterine capacity, litter size, and the physiological constraints that increase within-litter competition for limited uterine space and nutrient supply reducing piglet BW and increasing within-litter BW variation, which contributes to increased PWM. Moreover, as highlighted above there were dam line genetic differences in PWM, particularly in the third parity, were Landrace-sired dams had reduced PWM rate (<5.0%) compared to Yorkshire-sired dams, suggesting line-specific management adjustment could be beneficial for improving piglet survival. It is important to highlight the differences in herd parity structure between this study and typical U.S. commercial swine herds. In this study, the average parity was 2.3, whereas U.S. commercial herds typically target an average parity of 3.5 (Stalder et al., 2003). This difference in parity structure impacts key litter statistics, particularly PWM and NBA. For example, dams for the entire population in this study had a lower PWM (12.3%) and fewer piglets born alive (12.9 piglets) compared to current U.S. commercial herd benchmarks (15.4% PWM and 13.5 piglets, respectively; Miles et al., 2023), further illustrating the influence of parity on litter outcomes.

Finally, the current study demonstrated significant improvements to within-litter BW variation and PWM when utilizing reciprocal matings between breeds compared to intra-line matings. This suggests that heterosis effects from crossbreeding may help mitigate litter variability and piglet loss, underscoring the continued importance of strategic crossbreeding in commercial systems (Cassady et al., 2002; Moeller et al., 2004). Unfortunately, the unbalanced distribution of dam- to sire- line matings across parities as highlighted above, likely confounded the interactions between breed genetics and corresponding parity. This is further observed by the significant increase in litter size in intra-line matings (primarily third and fourth parity) compared to reciprocal matings (primarily first and second parity), so the parity influence may be greater than the heterosis effects of crossbreeding. Therefore, more targeted designed experiments to evaluate the influence of crossbreeding during later parities on litter size, piglet BW, within-litter variation, and PWM are needed to disentangle these complex relationships between genetics and parity.

In conclusion, the current study indicates that natural variation in early gestation maternal P4 levels in commercial herds exerts only a marginal direct influence on litter size, piglet BW, within-litter BW variation and PWM. Although limited direct relationships between P4, within-litter variation and PWM were observed, subtle differences in P4 between dam and sire genetics, particularly elevated P4 in Landrace-sired litters and corresponding increased piglet BW and decreased PWM, highlights possible indirect influences of P4 on within-litter variation and PWM that could improve piglet quality and survivability. Furthermore, established factors, such as within-litter BW variation, parity structure, and breed line interactions, remain the key drivers for farrowing success and piglet viability. These findings suggest that, rather than focusing on P4 variation alone, future management and genetic selection strategies should prioritize enhancing fetal growth uniformity by optimizing parity structure and maximizing the benefits of strategic crossbreeding to sustain improvements in piglet quality and survivability.

## Abbreviations:

BW: birth weight
BWCV: birth weight CV
CL: corpus luteum
MCR: metabolic clearance rate
NBA: number born alive
NBD: number born dead
OR: ovulation rate
P4: progesterone
PBA: piglets born alive
PND: piglets nursed by dam
PNM: pre- and peri-natal mortality
PWM: preweaning mortality
TB: total born
USMARC: U.S. Meat Animal Research Center
WW: weaning weight
WWCV: weaning weight CV
YLD: Yorkshire, Landrace, and Duroc
